# 150 Statewide Evaluation of CDC Priority Core Elements and NHSN SAAR Performance

**DOI:** 10.1017/ash.2026.10554

**Published:** 2026-06-23

**Authors:** Jocelyn Parrott

**Affiliations:** 1 Advocate Health

## Abstract

**Background:** Central line-associated bloodstream infections (CLABSIs) remain a significant source of preventable harm in hospitalized patients, often resulting from missed dressing changes and mislabeled tubing. Research indicates that most CLABSIs occur more than five days after catheter insertion, highlighting the importance of maintenance practices. This project, conducted at Atrium Health Union/Union West, aimed to reduce CLABSI rates by improving adherence to central line care protocols through innovative use of secure chat photography. **Methods:** Using the Plan-Do-Study-Act (PDSA) framework, the team identified procedural gaps in dressing changes and tubing labeling. A novel intervention was implemented: frontline nurses sent secure chat photos of central line dressings and tubing labels to leadership at each shift change via the electronic medical record (EMR). This real-time visual verification system enhanced compliance monitoring and accountability. Stakeholder engagement was prioritized through shared governance, pilot testing, and feedback loops. Pre- and post-intervention audits measured compliance, and statistical analysis validated the impact. **Results:** Pre-intervention compliance with central line maintenance protocols was 83%. Post-intervention, compliance improved to a range of 92.4% to 100% across seven units, averaging 95.5% with sustained results for a year thus far. This improvement correlated with a reduction in CLABSI rates, contributing to better patient outcomes and potential cost savings, as each CLABSI can cost between $16,000 and $45,000. The project is on track to meet its goal of ≥99% compliance, supported by ongoing audits, leadership feedback, and integration of the secure photo process into standard workflows. **Conclusion:** This initiative successfully leveraged digital tools to address a persistent clinical challenge. Secure photo documentation via EMR messaging proved to be an effective, scalable, and low-cost strategy for improving central line care compliance. The intervention not only enhanced patient safety and reduced infection risk but also promoted health equity by standardizing care across units. Its replicability across other healthcare settings makes it a valuable model for broader dissemination. The project demonstrates how technology-driven solutions can transform infection prevention practices and support institutional goals of safety, quality, and equity in care delivery.

